# The roles of post-translational modifications in the pathogenesis of RNA viruses: allies or adversaries?

**DOI:** 10.3389/fmicb.2026.1768721

**Published:** 2026-02-12

**Authors:** Tong Shao, Zhichao Pei, Yuting Wang, Yitong Zhao, Huimin Fan, Jiahui Pan

**Affiliations:** 1 Center of Translational Medicine, The Fourth Affiliated Hospital of Soochow University, Medical Center of Soochow University, Suzhou Dushu Lake Hospital, Suzhou, Jiangsu, China; 2 Key Laboratory of Vaccine, Prevention and Control of Infectious Disease of Zhejiang Province, Zhejiang Provincial Center for Disease Control and Prevention, Hangzhou, China; 3 Department of Cell Biology, School of Basic Medical Sciences, Harbin Medical University, Harbin, Heilongjiang, China

**Keywords:** pathogenesis, post-translational modifications, viral replication, RNA viruses, virus–host interaction

## Abstract

Infectious diseases continue to pose major threats to global public health, owing to the persistent emergence of novel and re-emerging viruses. However, the intricate mechanisms governing virus–host interactions remain incompletely understood. Precise regulation of protein function is critical during viral infection, and post-translational modifications (PTMs), as key modulators of protein activity, are extensively exploited by viruses at all stages of their life cycle. Upon entry into host cells, viruses frequently hijack host PTMs to reprogram cellular metabolism and signaling, thereby shaping infection outcomes. This review highlights recent advances in 10 major PTM types implicated in RNA virus infections, emphasizing their multifaceted roles across the viral life cycle. By integrating the latest proteomics findings, it aims to provide deeper insight into PTMs as potential targets for antiviral strategies and to explore their promise in treating virus-associated diseases.

## 1 Introduction

In recent years, the frequent occurrence of RNA virus infection has become an important focus of global public health (Mohanty et al., 2023). Numerous emerging, re-emerging, and rapidly spreading epidemics are closely associated with RNA virus infections (Chakrabartty et al., 2022), including the Severe acute respiratory syndrome coronavirus 2 (SARS-CoV-2) and influenza A virus (IAV) transmitted via respiratory routes, Ebola virus (EBOV) and Marburg virus (MARV) spread through direct contact, and arboviruses such as Dengue virus (DENV) and Zika viruses (ZIKV) transmitted by insect vectors. Moreover, several RNA viruses with notable zoonotic potential—such as Lassa virus (LASV), Rift Valley fever virus (RVFV), Rabies virus (RABV), and West Nile virus (WNV)—continue to pose threats at the human–animal interface. The host innate immune system triggers an antiviral immune response by rapidly recognizing pathogen-associated molecular patterns (PAMPs), which limits the replication and spread of the virus and provides essential signals for the initiation of adaptive immunity. However, almost all RNA viruses have evolved complex immune evasion strategies that interfere with host immune recognition and signal transduction mechanisms, thereby promoting sustained viral replication and enhancing pathogenicity (Naz et al., 2021). The interaction between viruses and hosts is central to determining infection severity and disease outcomes. Deep elucidation of the underlying molecular mechanisms is crucial for advancing effective antiviral strategies and developing innovative therapeutic approaches (Aw et al., 2025).

**Figure 1:**
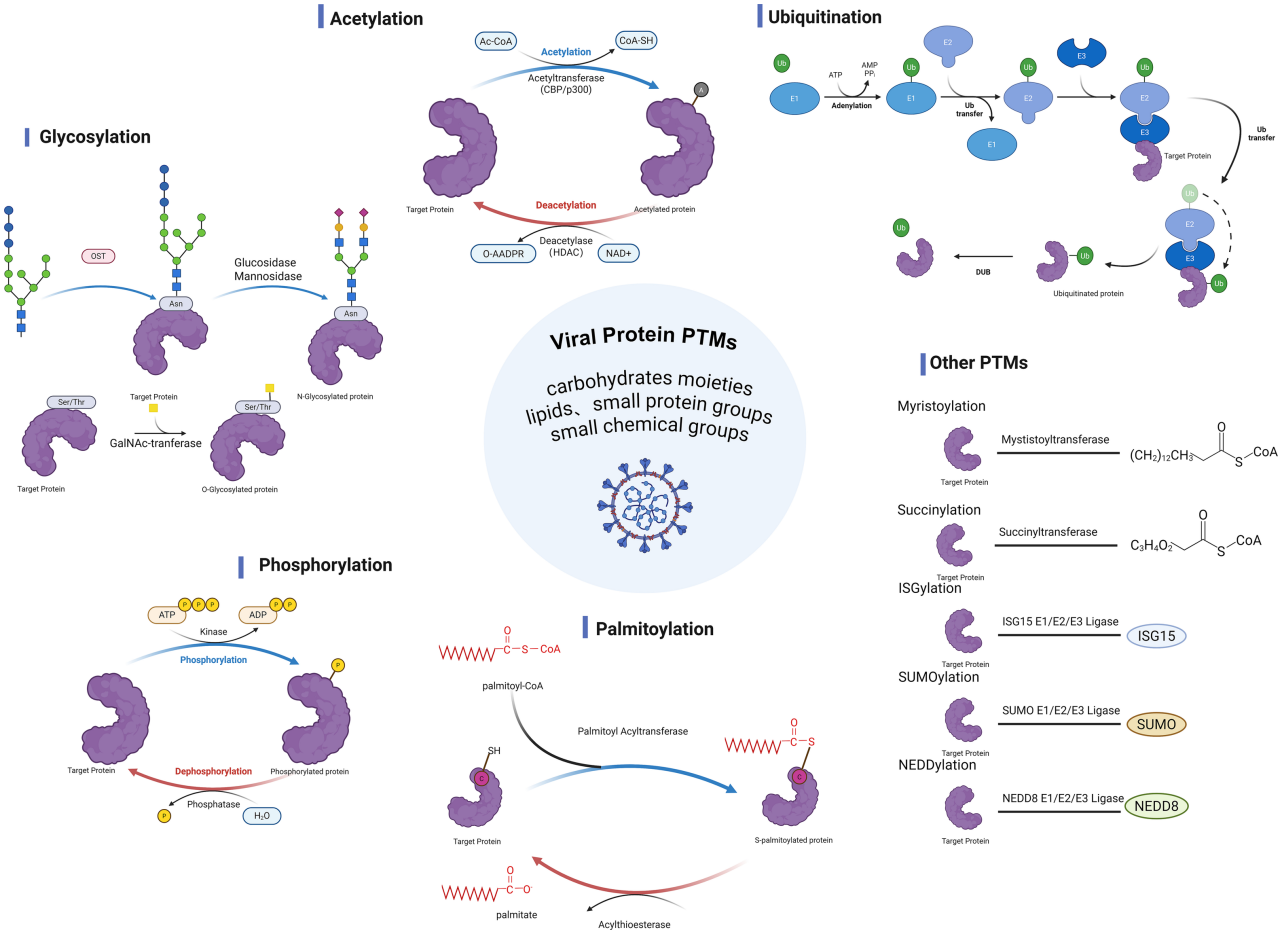
Post-translational modifications in viral infection and host response. During viral infection, various PTMs of viral proteins exert significant effects. These modifications include glycosylation, acetylation, ubiquitination, phosphorylation, and palmitoylation, as well as other types such as myristoylation, succinylation, SUMOylation, ISGylation, and NEDDylation. By altering the structure and function of viral proteins, these PTMs influence viral replication, assembly, and release, as well as host cell signaling and immune defense responses, thereby modulating the complex interactions between the virus and the host. Created with BioRender.com.

Post-translational modifications (PTMs) refer to enzymatic processes that add functional groups to proteins after translation, serving as a key mechanism for diversifying and finely tuning protein functions (Figure 1). To date, more than 680 distinct PTMs have been identified, such as phosphorylation, acetylation, and ubiquitination (Huang et al., 2025). With advances in detection technologies, novel PTMs such as palmitoylation, succinylation, and crotonylation have been increasingly discovered (Venne et al., 2014). During viral infection, viruses are highly dependent on host biomolecular mechanisms at all stages of their life cycle and frequently exploit the PTMs system to regulate the modification status of their own proteins, thereby enhancing antigenicity, replicative capacity, and pathogenicity (Cheng et al., 2022). In parallel, the host leverages PTMs to modify specific sites on viral proteins, shaping viral pathogenesis by activating immune response pathways and suppressing viral protein synthesis. Accumulating evidence indicates that PTMs do not act as isolated regulatory events, but are repeatedly redeployed as infection progresses, with distinct modification states supporting different functional requirements across the viral life cycle. This review focuses on 10 PTM types that capture this commonly investigated regulatory layer in RNA virus research—palmitoylation, myristoylation, glycosylation, phosphorylation, acetylation, succinylation, ubiquitination, SUMOylation, NEDDylation and ISGylation—with emphasis on their functional roles and underlying mechanisms in RNA virus infections.

## 2 Palmitoylation

Protein palmitoylation is a reversible process where palmitic acid is attached to certain cysteine residues of proteins through thioester bonds (Linder and Deschenes, 2007). Based on distinct modes of conjugation, palmitoylation can be categorized into O-palmitoylation, N-palmitoylation, and S-palmitoylation. Among these, S-palmitoylation is the most prevalent form and is regarded as the classical paradigm of protein palmitoylation. Palmitoylation dynamically regulates protein function and intracellular physiological roles by modulating hydrophobicity, conformational stability, and intracellular trafficking, thereby influencing subcellular localization and interactions with membrane structures (Li X. et al., 2022).

**Figure 2:**
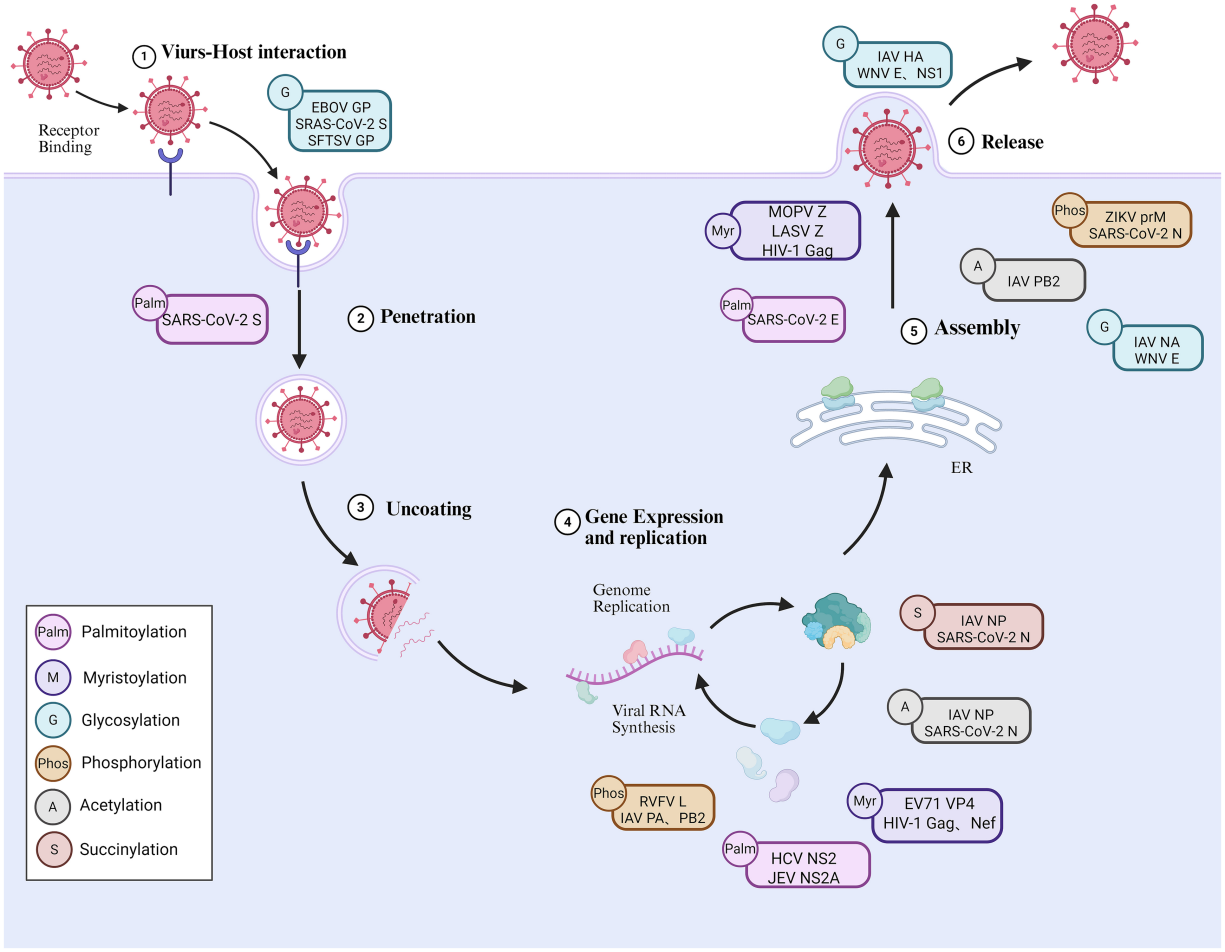
Multiple PTMs coordinate RNA viral infection processes throughout the viral life cycle. This figure illustrates PTMs occurring at different stages of the RNA virus life cycle. Glycosylation modifications of viral surface proteins (e.g., EBOV GP, SARS-CoV-2 S, and SFTSV GP) regulate receptor binding and viral entry processes, while palmitoylation enhance membrane binding and fusion efficiency. During replication, including phosphorylation, acetylation, succinylation, and myristoylation, regulate the activity and functional state of viral polymerase components and nucleoproteins. In later stages, PTMs on structural proteins support viral particle assembly, maturation, and release by synergistically influencing protein folding, trafficking, and intermolecular interactions. Different types of PTMs are represented by different colors symbols. Created with BioRender.com.

Palmitoylation supports multiple membrane-associated processes during RNA virus infection, including protein targeting, membrane fusion, particle assembly, and functional coordination of viral protein complexes (Figure 2 and Table 1). In 1979, researchers first identified palmitoylation of viral proteins in Sindbis virus (SINV) and Vesicular stomatitis virus (VSV) (Schmidt et al., 1979; Schmidt and Schlesinger, 1979). Most reported studies emphasize its role in facilitating infection, particularly among enveloped RNA viruses, where efficient membrane association is essential for viral propagation.

**Table 1:** The roles of post-translational modifications in viral pathogenesis.

Modification	Virus	Protein	Site	Function	References
Palmitoylation	SARS-CoV-2	S	C15	Promotes membrane fusion	Li D. et al. (2022)
	SARS-CoV-2	E	C40, C43, C44	Supports virion production	Wang et al. (2024)
	HCV	NS2	C113	Promotes NS2–NS3 autocleavage and regulates RNA replication	Wu et al. (2019)
	JEV	NS2A	C221	Enhances protein stability	Ma et al. (2023)
	ZIKV	E	C308	Reduces protein expression	Hu et al. (2023)
Myristoylation	LASV	Z	—	Promotes viral assembly and budding	Witwit et al. (2024)
	HIV-1	Gag	—	Promotes viral assembly	Guajardo-Contreras et al. (2024) and Zhou et al. (2023)
Glycosylation	SARS-CoV-2	S	N61, N122, N343	Reduces pseudovirus infectivity	Zhang F. et al. (2024), and Sztain et al. (2021)
	IAV	NA	N219	Reduces viral virulence	Bao et al. (2021)
	SFTSV	GP	—	Promotes viral attachment	Shimojima et al. (2024)
	EBOV	GP	N257, N563	Mediates viral attachment	Peng et al. (2022)
	RV	NSP4	—	Promotes cellular localization of viral proteins	Nurdin et al. (2023)
	WNV	E	—	Promotes virion release	Hanna et al. (2005)
	WNV	NS1	—	Promotes virus secretion	Whiteman et al. (2015)
	LASV	GPC	—	Suppresses host immune response	Zhu et al. (2021)
	HCoV-NL63	S	—	Enhances immune evasion capacity	Chmielewski et al. (2023)
Phosphorylation	RVFV	L	—	Reduces genome levels	Bracci et al. (2024)
	HMPV	P	—	Regulates the conformation of P protein	Thompson et al. (2023)
	IAV	PB2	S181	Regulates polymerase activity	Hu et al. (2024)
	IAV	PA	Y393, S395	Inhibits polymerase activity	Liu et al. (2023a)
	IAV	NS2	S23, S24, S25	vRNP nuclear export	Liu et al. (2025)
	IAV	M1	T108	vRNP nuclear export	Liu et al. (2023b)
	EBOV	VP30	S29	Inhibits viral transcription and virion packaging	Biedenkopf et al. (2016)
	EBOV	NP	T603	Inhibits viral transcription	Kamper et al. (2025)
	MARV	VP30	—	Inhibits viral transcription and viral growth	von Creytz et al. (2024)
	ZIKV	prM	Ser101, Thr107	Promotes production of infectious virions	Ren Y. et al. (2024)
	SARS-CoV-2	N	—	Balances genome replication and virion assembly	Syed et al. (2024) and Adly et al. (2023)
	HIV-1	Vif	T20	Enhances protein stability	Raja et al. (2022)
	SARS-CoV-2	N	Ser79	Enhances virion stability and infectivity	Ino et al. (2022)
Acetylation	IAV	NP	K77, K113, K229	Regulates viral replication. Affects viral genome packaging	Giese et al. (2017) and Ciminski et al. (2024)
	IAV	PB2	K187	Enhances viral pathogenicity	Hu et al. (2024)
	SARS-CoV-2	N	K61	Regulates the affinity between N protein and viral RNA	Hatakeyama et al. (2021)
Succinylation	SARS-CoV-2	N	—	Regulates protein dimerization	Liu Q. et al. (2022)
	IAV	NP	K87	Inhibits vRNP transport	Guillon et al. (2022)
Ubiquitination	IAV	NP	K184, K227, K273	Enhances viral replication efficiency	Lin et al. (2017)
	IAV	PB1	K578	Promotes polymerase dimerization	Gunl et al. (2023)
	IAV	M1	K242	Degrades the corresponding protein	Lin et al. (2023)
	IAV	NS2	K64, K88	Degrades the corresponding protein	Li et al. (2024)
	EBOV	VP35	K309	Enhances polymerase activity	Bharaj et al. (2017)
	SARS-CoV-2	N	K102, K347, K361	Enhances NP binding to the genome	Zhou et al. (2024)
	SARS-CoV-2	ORF7a	K119	Inhibits interferon signaling pathway	Cao et al. (2021) and Liu Z. et al. (2022)
	ZIKV	NS1	K265, K284	Degrades the corresponding protein	Huang et al. (2024)
	ZIKV	NS2A	K56	Inhibits endoplasmic reticulum autophagy	Zhang et al. (2024b)
SUMOylation	EBOV	VP24	—	Maintains protein stability	Vidal et al. (2019)
	IAV	NS1	K219, K70	Reduces the protein’s ability to inhibit the interferon signaling pathway	Santos et al. (2013)
	IAV	PB2	—	Weakens viral RNP complex activity	Wang G. et al. (2022)
	SARS-CoV-2	N	K65	Regulates protein nuclear translocation	Madahar et al. (2023)
Neddylation	EV71	VP2	K69	Promotes protein degradation	Wang H. et al. (2022)
	IAV	PB2	K699	Reduced protein stability	Zhang et al. (2017)
	IAV	M1	K187	Reduced protein stability	Li et al. (2020)
ISGylation	IAV	NS1A	K41	Suppresses protein function	Zhao et al. (2010), and Tang et al. (2010)
	SARS-CoV-2	N	K374	Inhibits protein oligomerization	Rhamadianti et al. (2024)
	HCV	NS5A	K20, K26, K44, K139, K166	Promotes RNA replication	Bawono et al. (2021)

### 2.1 Influences the membrane localization and fusion capability of viral proteins

Palmitoylation of viral proteins enhances their interaction with the plasma membrane, thereby promoting fusion between the viral and host cell membranes and increasing infection efficiency. Studies have shown that ZDHHC5 and GOLGA7 can bind to SARS-CoV-2 spike (S) protein and promote its palmitoylation process (Wu et al., 2021; Zeng et al., 2021). Several cysteine residues within the S protein—particularly Cys15 and those located in the C-terminal cytoplasmic tail—have been identified as critical palmitoylation sites. This modification is essential for S protein-mediated syncytia formation (Li D. et al., 2022; Mesquita et al., 2021). Furthermore, palmitoylation also promotes the proper trafficking of the mature S protein to the Golgi apparatus and plasma membrane (Tien et al., 2022), thereby triggering the fusion of virus and host cell membrane.

### 2.2 Influences the processes of viral assembly and particle maturation

Beyond viral entry, palmitoylation also contributes to viral assembly and particle maturation. The SARS-CoV-2 E protein is palmitoylated at residues C40, C43, and C44 by zDHHC3, 6, 12, 15, and 20. This modification is essential for the production of virus-like particles (VLPs) and for maintaining normal viral particle density (Wang et al., 2024), highlighting the role of palmitoylation in late structural stages of the viral life cycle.

### 2.3 Influences viral protein stability, abundance, and functionality

Palmitoylation can further modulate viral protein stability, abundance, and functional activity. In hepatitis C virus (HCV), palmitoylation of the NS2 protein at residue C113 facilitates RNA replication by promoting NS2–NS3 autolytic cleavage and recruiting the E2 protein to membrane microdomains (Wu et al., 2019). In Japanese encephalitis virus (JEV), palmitoylation of the NS2A protein at the C221 enhances protein stability, thereby increasing viral replication efficiency and virulence (Ma et al., 2023). In contrast, palmitoylation can also exert restrictive effects. ZDHHC11 interacts with ZIKV E protein and acts as the main catalytic enzyme for its palmitoylation. Overexpression of ZDHHC11 significantly reduces the level of ZIKV infection, whereas its knockdown enhances viral infectivity (Hu et al., 2023). The functional outcome of palmitoylation varies with the specific viral protein and host enzyme involved.

## 3 Myristoylation

Myristoylation is a covalent lipid modification in which myristic acid is attached to an amino acid residue of a protein, typically the glycine at its N-terminus. This process is catalyzed by N-myristoyltransferase (NMT), occurs co-translationally, and is considered irreversible (Bussienne et al., 2021). Myristoylation is widely utilized by RNA viruses and involves the attachment of a saturated C14 acyl chain that facilitates stable association of viral proteins with cellular membranes. Through this mechanism, it plays an important role in membrane targeting of viral proteins, thereby influencing key stages of the viral life cycle (Figure 2 and Table 1). This modification is broadly conserved across diverse virus families, highlighting a shared reliance on N-terminal lipidation for productive infection. Although most studies emphasize its infection-promoting roles, the dependence on host NMT activity also exposes myristoylation as a regulatory vulnerability that can be modulated to restrict viral propagation under specific conditions.

### 3.1 Modulates viral entry and genome replication

In nearly all picornaviruses, the capsid protein VP4 undergoes N-terminal myristoylation mediated by NMT within the host cell (Corbic Ramljak et al., 2018). For example, in enterovirus 71 (EV71), the N-terminus of VP4 is myristoylated, and although loss of this modification does not affect viral protein expression or virion morphology, it markedly reduces viral infectivity, underscoring its functional importance in early stages of infection. In retroviruses, myristoylation is typically highly conserved. With few exceptions, nearly all retroviral Gag proteins undergo co-translational myristoylation at the N-terminal glycine residue within the matrix (MA) domain (Wang et al., 2021). During human immunodeficiency virus type 1 (HIV-1) infection, myristoylated Gag and Nef proteins significantly enhanced viral replication and infectivity (Seaton and Smith, 2008). Collectively, these findings indicate that myristoylation promotes efficient viral entry and early replication by enabling dynamic membrane association and spatial organization of viral structural proteins across distinct virus families.

### 3.2 Influences the processes of viral assembly and particle maturation

Beyond viral entry, myristoylation plays a critical role in viral assembly and particle maturation. In mammalian viruses, the Z matrix protein and the stable signal peptide of the GP1/2 envelope glycoprotein are two of the four encoded proteins that undergo myristoylation (Carnec et al., 2024). The Z protein of Mopeia virus (MOPV) interacts with both N-myristoyltransferase 1 (NMT1) and 2 (NMT2), with NMT1 identified as the virus-dependent isoform that plays a dominant role in the production of infectious viral particles (Carnec et al., 2024). Consistent with this requirement, myristoyltransferase inhibitors such as DDD85646 block viral assembly and budding by inhibiting the myristoylation of the arenavirus Z protein and SSP. They also interfere with GP-mediated membrane fusion events (Witwit et al., 2024). These findings suggest that NMT-dependent myristoylation represents a potential broad-spectrum antiviral target against mammalian arenavirus infections.

In retroviruses, beyond myristoylation established role in promoting Gag multimerization and membrane association via the myristoyl switch in the HIV-1 MA domain, it may also regulate the ordered cleavage of Gag proteins in immature Mason–Pfizer monkey virus particles, thereby influencing viral maturation and infectivity (Castoralova et al., 2024). N-myristoylation of the Gag polyprotein is essential for its co-transport to late endosomes and lysosomes. Research has shown that inhibiting N-myristoylation of the Gag polyprotein significantly impairs HIV-1 assembly in macrophages and suppresses the formation of virus-containing compartments (VCCs) (Guajardo-Contreras et al., 2024). Furthermore, myristoylated Gag proteins form trimeric cores within membrane models and selectively bind dimeric RNA harboring packaging signals, providing a crucial structural basis for the initial assembly of the HIV-1 capsid (Zhou et al., 2023).

## 4 Glycosylation

Glycosylation can significantly change protein surface charge, spatial conformation, and stability, thereby modulating interactions with other biomolecules (He et al., 2024). Under the catalytic action of glycosyltransferases, glycan chains are efficiently and specifically transferred to designated sites on target proteins. Based on the mode of linkage between glycans and amino acid residues, glycosylation is primarily classified into two major types: O-glycosylation and N-glycosylation. N-glycosylation typically occurs at the Asn-X-Ser/Thr consensus sequence (where X represents any amino acid except proline), in which the glycan moiety is covalently attached to the amide nitrogen of asparagine residues (Pasala et al., 2024), whereas O-glycosylation predominantly takes place on serine or threonine residues, and the attached glycans are generally mucin-type oligosaccharides.

Different virus families exploit glycosylation in distinct ways to support infection and immune evasion (Figure 2 and Table 1). Flaviviruses rely on a site-restricted glycosylation pattern, in which the envelope (E) protein typically contains only one or two N-linked glycosylation sites (Carbaugh and Lazear, 2020). These individual glycans exert pronounced, site-specific effects on virion assembly and secretion, cell-type tropism, and receptor usage, including interactions with C-type lectin receptors such as DC-SIGN (Ishida et al., 2023; Liang et al., 2018). Because flaviviruses alternate between arthropod and vertebrate hosts, host-dependent differences in glycan processing further modulate glycan composition, allowing a limited number of glycosylation sites to function as adaptable determinants of infectivity. In contrast, coronaviruses adopt an extensive, surface-distributed glycosylation strategy, most prominently on the spike (S) protein, which contains numerous N-glycosylation sites that collectively form a dense glycan layer (Watanabe et al., 2020; Grant et al., 2020). This glycan layer supports protein folding and conformational stability while shaping antibody accessibility and immune recognition (Zhang F. et al., 2024). The position-dependent differences in glycan maturation highlight how the overall glycosylation pattern, rather than a single site, plays a dominant role in the coronavirus-host interaction.

### 4.1 Modulates viral replication and infectivity

Glycosylation frequently acts at early stages of infection, yet the degree of dependence on specific glycan sites varies markedly across virus families and even among viral variants. In SARS-CoV-2, removal of N-linked glycans at the N61 and N122 sites of the spike protein markedly reduces pseudovirus infectivity, indicating a general requirement for glycosylation at these positions (Zhang F. et al., 2024). By contrast, the contribution of glycosylation at other sites depends on the viral genetic background, as illustrated by differential effects of N717 and N801 glycan loss across SARS-CoV-2 variants (Watanabe et al., 2020). Loss of glycosylation at these sites nearly abolishes infectivity in the Omicron BA.1 variant, whereas the D614G variant remains largely unaffected under the same conditions (Lusvarghi et al., 2023). These findings illustrate that glycosylation can be reshaped during viral evolution to accommodate structural changes in surface proteins.

IAV has two main surface glycoproteins, haemagglutinin (HA) and neuraminidase (NA). NA functions during the late stages of viral replication by catalyzing the cleavage of the linkage between sialic acid and HA, thereby facilitating the release of progeny virions. The N-glycosylation modification of NA protein regulates the budding, replication ability and virulence of the virus. The N-glycosylation site at position 219 is particularly critical, and its mutation can significantly reduce the virulence of the virus and can be used to develop attenuated vaccines (Bao et al., 2021; Ostbye et al., 2020).

### 4.2 Regulates the ability to bind to host cells

For many enveloped viruses, glycosylation of surface proteins plays a critical role in shaping receptor usage and attachment efficiency. Severe fever with thrombocytopenia syndrome virus (SFTSV) exploits N-linked glycosylation on its glycoprotein (GP) to mediate specific interactions with host C-type lectins, thereby facilitating viral attachment and promoting infection (Shimojima et al., 2024). Similarly, the glycoprotein of EBOV contains multiple complex N- and O-linked glycosylation sites, among which N-linked glycans at positions N257 and N563 are critical for DC-SIGN-mediated viral attachment (Peng et al., 2022; Bagdonaite et al., 2024).

In SARS-CoV-2, glycosylation of the S protein critically shapes receptor engagement. Glycosylation at N165 and N234 stabilizes the conformation of the receptor-binding domain (RBD), thereby directly regulating its interaction with the host ACE2 receptor and influencing viral infectivity. Additionally, the N-linked glycan at N343 has been implicated in facilitating the transition of the RBD from a “closed” to an “open” conformation, a prerequisite for receptor engagement (Sztain et al., 2021). These examples illustrate how glycosylation modulates viral entry not merely by shielding epitopes, but by actively shaping protein conformations that enable receptor engagement.

### 4.3 Maintains the structural stability of viral protein folding, facilitating viral particle release

Beyond entry, glycosylation is essential for maintaining the structural integrity of viral proteins and ensuring efficient virion assembly and release. In rotavirus (RV), defects in glycosylation result in aberrant intracellular localization of the nonstructural protein NSP4, thereby disrupting calcium ion homeostasis, impairing viroplasm formation, and reducing viral replication efficiency. These effects vary across different cell types, with only mild inhibition of replication observed in MA104 cells, but a marked impairment of viral assembly in HT29 cells. Animal studies further demonstrated that viruses lacking NSP4 glycosylation exhibit attenuated virulence in mice, highlighting the essential regulatory role of NSP4 glycosylation in viral pathogenicity (Nurdin et al., 2023). In addition, deglycosylation of the WNV envelope protein similarly results in reduced release of mature viral particles (Hanna et al., 2005).

### 4.4 Mediates viral immune evasion

Glycosylation can promote viral immune evasion through the formation of a “glycan shield” that masks neutralizing epitopes on the viral surface. The glycoprotein complex (GPC) of LASV contains 11 N-glycosylation sites, among which N99, N119, N167, N365, and N373 are believed to conceal epitopes on GPC and suppress host immune responses (Zhu et al., 2021). In arenaviruses, the removal of glycans at N83 and N166 on the GPC protein disrupts the glycan shielding effect, thereby enhancing viral immunogenicity and increasing sensitivity to neutralizing antibodies, ultimately weakening viral pathogenicity (Koma et al., 2021). Moreover, for SARS-CoV-2, glycosylation of the ORF8 protein reduces its binding capacity to immune cells, demonstrating that glycosylation is involved in immune evasion (Wu et al., 2023). In contrast, the S protein of human coronavirus NL63 (HCoV-NL63) exhibits dense oligomannose-type glycosylation, forming a more effective glycan shield and further enhancing its capacity to evade immune detection (Chmielewski et al., 2023). Such glycan-based masking strategies allow viruses to modulate immune visibility without altering underlying protein sequences, providing a flexible means of immune evasion across diverse host environments.

## 5 Phosphorylation

Protein phosphorylation is a reversible process, catalyzed by protein kinases and reversed by protein phosphatases. Specifically, protein kinases mediate the transfer of a phosphate group (PO₄) from ATP to the hydroxyl group of serine, threonine, or tyrosine residues on target proteins, whereas protein phosphatases reverse this modification by hydrolyzing the phosphoester bond, thereby removing the phosphate moiety and restoring the unmodified state (Ardito et al., 2017). Protein phosphorylation is essential for the successful infection cycle of many intracellular pathogens (Figure 2 and Table 1). In cytoplasm-replicating non-segmented negative-strand RNA viruses, phosphorylation predominantly targets polymerase cofactors, thereby indirectly shaping RdRp activity (Das and Pattnaik, 2004; Sugai et al., 2012). In contrast, in positive-strand RNA viruses such as flaviviruses and coronaviruses, phosphorylation is primarily associated with enhancing the enzymatic activity of viral proteins and promoting viral genome replication (Boytz and Laurent-Rolle, 2024). For nuclear-replicating RNA viruses exemplified by influenza virus, polymerase function is further intertwined with host transcriptional regulatory networks, rendering phosphorylation a potentially more direct determinant of transcription–replication balance (Dawson et al., 2020).

### 5.1 Modulates viral genome replication

Efficient viral genome replication depends on finely coordinated, reversible phosphorylation of viral polymerase components and their cofactors, allowing different functional states to operate at distinct stages of the replication cycle. The L protein of RVFV is regulated through a dynamic phosphorylation–dephosphorylation cycle mediated by CK1α kinase and PP1α phosphatase; inhibition of either enzyme leads to a marked reduction in viral genomic RNA levels, highlighting the requirement for balanced phosphorylation states during replication (Bracci et al., 2024). In human metapneumovirus (HMPV), the multifunctional regulatory role of phosphorylation on the polymerase cofactor P protein has also been established (Thompson et al., 2023). This study demonstrated that several phosphorylation sites of the P protein are located within intrinsically disordered regions (IDRs). Either phosphomimetic substitution or dephosphorylation mutations at specific sites (e.g., S268 and S271) significantly impair viral RNA synthesis, inclusion body (IB) formation, and viral rescue efficiency. The inability of some phosphomimetic P mutants to restore minireplicon activity suggests a requirement for multiple P protein populations existing in distinct phosphorylation states to coordinate efficient RNA synthesis throughout the viral replication cycle.

Phosphorylation at the S181 site of IAV PB2 negatively regulates IAV replication and pathogenicity. The phosphorylation-mimetic mutation (S181E) results in reduced polymerase activity, restricted viral replication, attenuated virulence, and diminished expression of immune-related factors, suggesting that dynamic phosphorylation at this site is required to maintain the proper function of PB2 (Hu et al., 2024). The degree and timing of phosphorylation may directly influence the balance of the viral life cycle, with hyperphosphorylation impairing efficient replication and transmission. Phosphorylation at residues Y393 and S395 of the IAV PA subunit also suppresses polymerase activity by weakening its binding affinity to vRNA and cRNA, thereby reducing viral replication efficiency (Liu et al., 2023a). The replication of IAV is also dependent on phosphorylation at the T108 residue of the M1 protein. Loss of phosphorylation at this site prevents M1 from entering the nucleus and impairs its ability to mediate nuclear export of vRNPs (Liu et al., 2023b). Phosphorylation of NS1 at S205 further modulates viral replication by promoting its interaction with the host restriction factor DDX21, thereby enhancing polymerase activity (Patil et al., 2021).

### 5.2 Modulates viral genome transcriptional

Phosphorylation also serves as a key regulatory switch in viral transcription by controlling the activity of viral transcription factors. The transcription process of EBOV is tightly regulated through dynamic phosphorylation of the viral transcription factor VP30 by host phosphatases, with the phosphorylation status of VP30 and NP playing a critical role in this regulation. The N-terminal domain of VP30 harbors a dynamically phosphorylated site that directly modulates its interaction with viral RNA and NP (Biedenkopf et al., 2016). Studies have demonstrated a dual role of VP30 phosphorylation: phosphorylated VP30 exhibits reduced binding affinity to viral RNA and NP, thereby inhibiting the initiation of viral transcription and virion packaging, whereas dephosphorylation of VP30 is essential for viral genome transcription (Modrof et al., 2002). Concurrently, the phosphorylation status of NP itself—particularly the dynamic phosphorylation at residue T603—serves as an important regulatory element in viral transcription. Dephosphorylation at NP-T603 promotes transcription initiation, while its phosphorylated state may exert an inhibitory effect (Kamper et al., 2025). A similar transcriptional regulation mechanism has been also observed in MARV, wherein NP recruits PP2A to efficiently promote VP30 dephosphorylation. Induction of VP30 hyperphosphorylation markedly impairs viral transcription and propagation, underscoring the importance of phosphatase-mediated regulation in filovirus transcriptional control (von Creytz et al., 2024).

### 5.3 Affects viral particle maturation and release

During ZIKV infection, the expression of PIM1 kinase is significantly upregulated in host cells and mediates the phosphorylation of the prM protein. This modification disrupts the interaction between prM and the E3 ubiquitin ligase AMFR, thereby preventing AMFR-mediated ubiquitination and degradation of prM and promoting virion maturation (Ren Y. et al., 2024). The N protein of SARS-CoV-2 is extensively phosphorylated and displays distinct functional roles at different stages of infection. Lower levels of N phosphorylation favor viral particle assembly, whereas higher phosphorylation levels enhance viral genome replication (Syed et al., 2024). Phosphorylation of the serine-rich (SR) region interferes with RNA binding by disrupting intramolecular interactions and introducing electrostatic repulsion between phosphorylated residues and the RNA-binding domain (Adly et al., 2023). This dual role highlights an intrinsic regulatory mechanism during infection, wherein the virus balances efficient genome replication with effective virion assembly to maximize transmissibility and adaptability.

### 5.4 Impacts the regulation of host antiviral signaling pathways

In addition to directly modifying viral proteins, phosphorylation modifications can also facilitate viral infection by modulating host proteins. In enteroviruses, phosphorylation of the highly conserved Ser/Thr125 residue of 2A^pro^ activates its proteolytic activity against the eukaryotic initiation factor 4GI, thereby enhancing the adaptability of enteroviruses to the host and ensuring viral proliferation (Wang et al., 2023). HIV-1 phosphorylates the Vif protein via the Tat-activated AKT signaling pathway, which increases Vif stability and enables more efficient targeting and degradation of the host antiviral factor APOBEC3G, thereby attenuating its potent antiviral activity (Raja et al., 2022). Similarly, phosphorylation of the SARS-CoV-2 nucleocapsid protein at Ser79 promotes its interaction with the host protein Pin1, and this interaction enhances the infectivity of viral particles (Ino et al., 2022).

## 6 Acetylation

Acetylation refers to the process by which acetyl groups are transferred to specific amino acid residues in proteins in response to acetyltransferases. This modification that occurs in two forms, one is an N-terminal acetylation modification, and the other is an acetylation modification that occurs on lysine (Verdin and Ott, 2015). N-terminal acetylation is highly prevalent in eukaryotes, occurring in approximately 85% of eukaryotic proteins during translation, and is considered an irreversible modification. In contrast, lysine acetylation takes place on the *ε*-amino group of lysine side chains and is a dynamic, reversible process. It is tightly regulated by the coordinated actions of acetyltransferases and deacetylases and plays vital roles in various biological processes (Serman et al., 2023). During infection, acetylation can influence viral infection by altering protein stability, localization, enzymatic activity, and protein–protein or protein–RNA interactions (Figure 2 and Table 1).

Phosphorylation and acetylation have been reported to target the same viral proteins during RNA virus infection. Rather than acting as isolated regulatory events, these modifications are deployed at different stages of infection to support distinct functional requirements of viral proteins. In influenza A virus, the nucleoprotein (NP) undergoes acetylation at multiple lysine residues that supports viral RNA synthesis, whereas phosphorylation of NP at serine and threonine residues regulates its intracellular trafficking and ribonucleoprotein remodeling during infection (Giese et al., 2017; Hu et al., 2020). Distinct post-translational modifications can be differentially deployed on the same viral protein to support stage-specific functional requirements across the viral life cycle.

### 6.1 Modulates viral entry and genome replication

Acetylation has emerged as an important regulatory layer in IAV infection, reshaping how the nucleoprotein (NP) engages viral RNA (vRNA) and how genome segments are coordinated, with direct consequences for viral replication output. Several proteins within the IAV ribonucleoprotein (RNP) complex are subject to acetylation, which in turn modulates viral transcription and replication. Acetylation at lysine 229 (K229) of NP is associated with efficient production of infectious viral particles (Giese et al., 2017). Moreover, the coordinated packaging of the IAV genome depends on the specific interaction between the RNA-binding groove of NP and the vRNA packaging signals—an interaction regulated by the acetylation of lysine residues (Ciminski et al., 2024).

### 6.2 Influences the processes of viral assembly and particle maturation

Acetylation directly modulates the function of key IAV proteins, influencing viral particle production and pathogenicity. The K187 residue of the PB2 subunit in the IAV polymerase undergoes acetylation. This site is located on the surface of the PB2 protein and is highly conserved. Acetylation at K187 stabilizes the PB2 subunit and enhances the pathogenicity of the virus (Hu et al., 2024). On the other hand, N-terminal acetylation of the PA-X protein is essential for IAV-mediated host shutoff (Oishi et al., 2018). Subsequent research has identified two acetylation sites within PA-X. While acetylation at either site is sufficient to ensure proper nuclear localization, only acetylation at the initiating methionine is critical for the full execution of its host shutoff function (Daly et al., 2026). This separation between localization competence and effector potency highlights how acetylation can partition viral protein functions across infection stages.

### 6.3 Alters viral protein stability and activity

Acetylation can significantly impact the enzymatic activity of viral proteins, and its functional direction can vary with both residue context and host enzyme identity. The flavivirus NS3 protein, a highly conserved multifunctional enzyme, possesses serine protease activity at its N-terminus and SF2-family helicase activity at its C-terminus—both of which are indispensable for viral replication. The host acetyltransferase KAT5γ acetylates NS3 to modulate its helicase function, thereby facilitating efficient viral replication (Serman et al., 2023).

In IAV, NP acetylation involves multiple host acetyltransferases, and perturbing these enzymes can produce distinct effects on viral polymerase activity. Silencing the histone acetyltransferases PCAF via RNA interference enhances viral polymerase activity, whereas silencing GCN5 reduces polymerase activity and suppressing viral replication (Hatakeyama et al., 2018). Similarly, acetylation at the K19 site of the PA subunit enhances the activity of the viral endonuclease and RNA polymerase (Hatakeyama et al., 2022). These observations suggest that “acetylation status” alone is insufficient to predict functional impact—enzyme context and the specific acetylation site are often key determinants that shape the infection outcome.

### 6.4 Affects the interactions between the virus and other molecules

Acetylation can enhance the interactions of viral proteins with host factors or viral RNA, thereby supporting efficient viral infection. The E protein of SARS-CoV-2 interacts with BET proteins (particularly the second bromodomain of BRD4) via acetylation, leading to the suppression of the host antiviral response (Chen et al., 2022). *In vitro* studies have identified 12 acetylated lysine residues on the SARS-CoV-2 N protein. Among them, acetylation at K61 was shown to modulate the binding affinity between the N protein and viral RNA (Hatakeyama et al., 2021).

## 7 Succinylation

Succinylation is characterized by the covalent attachment of a succinyl group, typically donated by succinyl-CoA, to lysine residues of proteins. This modification not only alters the spatial conformation of the protein due to the introduction of the succinyl group but also, by changing the charge of the lysine residue, further influences the physicochemical properties and functional alterations of the protein (Hou et al., 2024). Through these physicochemical effects, succinylation suggests a potential regulatory role in viral replication processes (Figure 2 and Table 1).

In SARS-CoV-2, succinylation has been detected on both structural and nucleocapsid proteins during infection. For instance, at 24 h post-infection, high levels of succinylation were detected on the M and N proteins. Further analysis showed that the cytoplasmic domain of the M protein contains two succinylation sites, while the N protein harbors 12 such sites. Two of the succinylation sites (K65 and K102) are located within the RNA-binding domain of the N protein, while the remaining 10 are found within or near the dimerization domain. These modifications may affect N protein dimerization, thereby promoting viral replication and infection (Liu Q. et al., 2022).

In contrast, succinylation can also exert restrictive effects on viral propagation, the NP of IAV undergoes succinylation at the highly conserved K87 residue. This modification alters the electrostatic interactions between NP and viral RNA, thereby affecting the transport of vRNPs and ultimately hindering efficient viral replication and spread (Guillon et al., 2022).

## 8 Ubiqutination

Ubiquitin (Ub) is a highly conserved 76-amino-acid polypeptide ubiquitously expressed, underscoring its fundamental role in diverse cellular processes. Ubiquitination refers to the covalent attachment of ubiquitin to substrate target proteins through a highly coordinated enzymatic cascade involving E1 activating enzymes, E2 conjugating enzymes, and E3 ligases, among which E3 ligases confer substrate specificity (Zheng and Shabek, 2017). The type of ubiquitin chain linkage determines the direction of the modified protein: polyubiquitin chains linked through lysine 48 (K48) predominantly target substrates for proteasome-dependent degradation, whereas chains linked through lysine 63 (K63) are primarily associated with endocytosis, intracellular trafficking, and the modulation of enzymatic activity (Kulathu and Komander, 2012).

**Figure 3:**
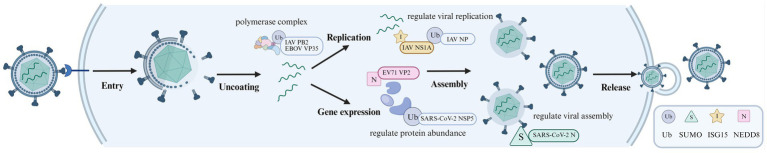
Roles of ubiquitin and ubiquitin-like modifications across the viral life cycle. Upon viral entry and uncoating, the viral genome is released into the cytoplasm, where the viral polymerase complex initiates genome replication and gene expression. Ubiquitination and multiple ubiquitin-like modifications participate in distinct phases of the viral life cycle. Ubiquitination acts upon the viral polymerase complex and other viral proteins, influencing replication-associated processes and regulating viral protein stability to control viral replication. SUMOylation may participate in viral particle assembly and inhibit replication in certain viruses. ISGylation and NEDDylation exert differing effects on viral infection by influencing viral protein stability or promoting their degradation. Created with BioRender.com.

Given its central regulatory role, ubiquitination is tightly controlled by deubiquitinating enzymes (DUBs), which remove ubiquitin moieties from substrates and maintain the dynamic balance of intracellular ubiquitin signaling (Lange et al., 2022). As a critical modulator of host–virus interactions, ubiquitination orchestrates innate immune signaling and viral pathogenic mechanisms, exhibiting a dualistic role that can either facilitate or inhibit viral infection (Figure 3 and Table 1). In many cases, the direction of ubiquitin signaling during infection reflects a competition over three linked control points: which E3 ligase accesses the substrate, what chain topology is installed, and whether deubiquitinases remove the mark before proteasomal delivery (Dugied et al., 2024). The types of ubiquitin chains formed, the specific modification sites, and the balance between E3 ligases and deubiquitinases all determine that ubiquitination supports viral replication or contributes to host restriction.

### 8.1 Affects viral genome replication and transcription

Following entry and uncoating, RNA viruses must rapidly establish efficient genome replication and transcription to gain a replicative advantage within the host cell. During the early phase of infection, ubiquitination often acts in a non-degradative capacity, shaping viral replication complex formation and supporting replication-associated activities. Influenza A virus (IAV) provides a representative example, all components of the ribonucleoprotein complex (RNP) are subject to ubiquitination (Kirui et al., 2016). During IAV infection, the E3 ubiquitin ligase CRL4 mediates non-degradative ubiquitination at lysine 29 (K29) of the PB2 subunit, markedly enhancing viral replication efficiency and infectivity (Karim et al., 2020). Furthermore, nucleoprotein (NP) undergoes CNOT4-mediated monoubiquitination at multiple sites, which facilitates viral RNA replication (Lin et al., 2017). Specific ubiquitination of the PB1 subunit at lysine 578 (K578) promotes polymerase dimerization and NP binding, thereby augmenting the activity of the viral polymerase complex (Gunl et al., 2023). These studies illustrate how IAV employs precise, non-degradative ubiquitination to optimize polymerase function and replication.

A similar regulatory paradigm is observed in other RNA viruses. VP35 serves as a key cofactor of the EBOV polymerase complex (Basler et al., 2003; Cardenas et al., 2006). It has been demonstrated that VP35 interacts with the host E3 ubiquitin ligase TRIM6, undergoing site-specific ubiquitination. TRIM6 not only modulates VP35 function but also enhances its polymerase activity, thereby increasing viral replication efficiency (Bharaj et al., 2017). In SARS-CoV-2, the nucleocapsid (N) protein is modified by K29-linked polyubiquitination at lysine residues K102, K347, and K361. This modification strengthens NP binding to the viral genome, facilitating viral proliferation (Zhang F. et al., 2024). Together, these studies illustrate how viruses employ precise, non-degradative ubiquitination to rapidly assemble functional replication complexes and initiate robust genome replication and transcription.

### 8.2 Affect host antiviral mechanisms

As viral replication progresses, host antiviral defenses such as innate immune signaling, autophagy, and apoptosis are activated to restrict viral spread. At this stage, viruses increasingly exploit ubiquitination-dependent pathways to modulate host antiviral mechanisms and maintain a cellular environment permissive for continued replication (Chen et al., 2023). ZIKV non-structural protein NS2A is ubiquitinated through K48 chain mediated by E3 ubiquitin ligase AMFR and subsequently binds to ER autophagy receptor FAM134B, promoting its degradation. This process suppresses ER-phagy, induces ER stress, and ultimately enhances viral infectivity and pathogenicity (Zhang et al., 2024b).

During SARS-CoV-2 infection, lysine residue 119 of ORF7a protein is ubiquitinated, which subsequently inhibits STAT2 phosphorylation and suppresses the interferon-*α* signaling pathway, facilitating viral evasion of host immune surveillance (Cao et al., 2021). Beyond immune suppression, ubiquitination of ORF7a also contributes to fine-tuning host cell fate. ORF7a recruits the anti-apoptotic protein BclXL to the endoplasmic reticulum, thereby activating the PERK-eIF2α-CHOP pathway, inducing ER stress-mediated apoptosis. However, ubiquitination of ORF7a disrupts its interaction with BclXL, reducing its accumulation in the endoplasmic reticulum, which lowers ER stress levels and partially mitigates apoptosis. This dynamic modulation of ORF7a ubiquitination illustrates how viruses balance immune suppression and host cell survival to sustain productive infection (Liu Z. et al., 2022). Through these mechanisms, ubiquitination emerges as an important means by which viruses attenuate host antiviral barriers to sustain productive infection.

### 8.3 Ubiquitin–deubiquitinase control of viral protein stability

At later stages of infection, precise control of viral protein abundance becomes essential to coordinate replication, assembly, and egress. Ubiquitination and deubiquitination jointly regulate the stability of viral proteins, shaping their fate at different stages of infection. Numerous studies have demonstrated that host E3 ubiquitin ligases specifically mediate the ubiquitination of viral proteins, triggering their degradation. For instance, WWP2 targets lysine residues Lys265 and Lys284 on the ZIKV NS1 protein, mediating K63- and K48-linked ubiquitination respectively, which leads to NS1 degradation and consequently inhibits ZIKV infection (Huang et al., 2024). Similarly, in IAV, the E3 ligase TRIM21 recognizes the M1 protein at residue R95, induces ubiquitination at lysine 242 (K242), and promotes its proteasomal degradation, thereby attenuating viral replication and pathogenicity (Lin et al., 2023).

In coronaviruses, ubiquitin-mediated degradation of viral proteins constitutes an important antiviral mechanism, which viruses actively counteract to preserve essential factors. In SARS-CoV-2, the viral proteins NSP5 and ORF6 are targeted by distinct host E3 ligases for ubiquitin-mediated degradation, thereby impairing their anti-interferon functions (Zhou et al., 2024b; Zhang et al., 2024a). Conversely, SARS-CoV-2 exploits deubiquitination to stabilize key viral proteins. The ORF9b protein undergoes ubiquitination and proteasomal degradation; however, the deubiquitinating enzyme USP29 removes ubiquitin chains from ORF9b, preventing its degradation and enhancing protein stability (Gao et al., 2022; Yu et al., 2024). At the same time, ORF9b is subject to K48-linked polyubiquitination at lysine 67 (K67) mediated by CUL5, which restricts viral replication (Zhou et al., 2024a). This restriction is counteracted by the binding of HSP90α to ORF9b, forming a CUL5–TOM70–HSP90α regulatory complex that preserves ORF9b function during infection.

Ubiquitin-dependent control of viral protein abundance can also vary across different stages of infection. Studies have revealed that the expression level of IAV NS2 protein exerts a dual effect on the viral life cycle: during the early phase of infection, low abundance of NS2 facilitates viral transcription, whereas elevated levels of NS2 inhibit transcription while simultaneously promoting viral RNA synthesis (Guillon et al., 2022). Furthermore, NS2 undergoes polyubiquitination at lysine residues K64 and K88, targeting it for proteasomal degradation. Nonetheless, this ubiquitination process does not impair NS2-mediated viral ribonucleoprotein (vRNP) nuclear export. The host deubiquitinating enzyme OTUB1 removes the polyubiquitin chains conjugated to NS2, markedly enhancing its stability and promoting its accumulation during the late stages of viral infection. This indicates that the virus prolongs NS2’s half-life by modulating host deubiquitinase activity to fulfill stage-specific functional requirements (Li et al., 2024). Through this dynamic regulation of ubiquitination and deubiquitination, NS2 protein levels are adjusted to meet stage-specific functional demands, illustrating how protein degradation and stabilization together shape antiviral restriction while allowing selected viral factors to persist when required.

## 9 Ubiquitin-like protein modification

### 9.1 SUMOylation

Small ubiquitin-like modifiers (SUMOs) represent a crucial class of protein post-translational modification molecules. The covalent attachment of a SUMO protein to lysine residues on target proteins is referred to as SUMOylation (Martin et al., 2007). Many SUMOylated proteins also harbor SUMO interaction motifs (SIMs), which mediate non-covalent interactions with free SUMO or SUMOylated proteins, thereby broadening their functional regulatory scope.

SUMOylation plays a crucial role in facilitating viral replication and modulating host immune defenses (Figure 3 and Table 1). The envelope and precursor membrane (prM) proteins of DENV are identified as potential SUMOylation substrates. Their SUMOylation facilitates viral replication and transmission in *Aedes aegypti* (Weng and Shiao, 2022). SARS-CoV-2 N protein regulates nuclear translocation and viral particle assembly through SUMO modification (Madahar et al., 2023). Interfering with N protein SUMOylation can significantly reduce the efficiency of viral replication, reflecting its potential as a therapeutic target (Ren J. et al., 2024). Additionally, VP24 of EBOV is a well-characterized SUMOylation target. This modification maintains the protein’s stability and enhances its capacity to suppress type I interferon signaling and block STAT1 nuclear translocation. Loss of SUMOylation results in reduced VP24 stability and diminished binding affinity to the nuclear import receptor KPNA5, thereby impairing its immune antagonistic function (Vidal et al., 2019).

Notably, SUMOylation does not uniformly favor viral infection. In certain contexts, SUMO modification can restrain viral replication by dampening the activity of key viral proteins. SUMOylation of the IAV NS1 protein attenuates its capacity to inhibit interferon signaling (Santos et al., 2013), while SUMOylation of the PB2 subunit compromises the activity of the viral RNP complex, thereby reducing viral replication efficiency and virulence (Wang G. et al., 2022).

Ubiquitination and SUMOylation frequently converge on the same viral proteins and operate in a coordinated or sequential manner to regulate viral protein localization, stability, and activity. In IAV, a representative example of such interplay is observed in the matrix protein M1. During infection, M1 can undergo both ubiquitination and SUMOylation at overlapping lysine residues, and host factors facilitate a functional shift from ubiquitination to SUMOylation, promoting efficient nuclear export of viral ribonucleoproteins and supporting productive replication (Gao et al., 2015). Beyond direct modification switching, parallel regulation by ubiquitination and SUMOylation has also been documented for viral proteins involved in genome replication. The NP of IAV is subject to both SUMOylation and ubiquitination during infection, with these modifications contributing to RNP activity and viral RNA synthesis (Liao et al., 2010). Although these modifications do not necessarily compete for the same residues, their convergence on shared viral factors facilitates transitions between nuclear trafficking, genome replication, and particle assembly across the viral life cycle.

### 9.2 NEDDylation

NEDDylation is a ubiquitin-like post-translational modification in which the neural precursor cell expressed developmentally downregulated protein 8 (NEDD8) is covalently conjugated to lysine residues of substrate protein. Owing to its substantial sequence similarity to ubiquitin, NEDDylation follows an enzymatic cascade analogous to ubiquitination and often targets lysine residues that are also subject to other post-translational modifications, thereby allowing potential competition or coordination among distinct regulatory pathways. NEDD8 is typically conjugated to lysine residues, which are also potential targets for other modifications, indicating possible competition or crosstalk among different post-translational modifications.

Several viral proteins have been reported as targets of neddylation (Figure 3 and Table 1). The VP2 protein of EV71 undergoes neddylation at lysine 69 (K69), a modification that facilitates the degradation of viral proteins and consequently suppresses EV71 proliferation (Wang H. et al., 2022). The PB2 and M1 proteins of IAV are subject to neddylation, which reduces protein stability and leads to diminished viral replication capacity (Zhang et al., 2017; Li et al., 2020).

### 9.3 ISGylation

ISG15 is a 17-kDa ubiquitin-like protein that is robustly induced in response to viral infection. Conjugation of ISG15 to target proteins, termed ISGylation, occurs through an enzymatic cascade that parallels ubiquitination and results in the covalent attachment of ISG15 to specific lysine residues on substrate proteins (Mustachio et al., 2018). ISGylation serves as a host antiviral mechanism by impairing the function of viral proteins and restricting viral replication (Figure 3 and Table 1). In IAV, ISGylation of the NS1A protein leads to its functional loss, thereby suppressing viral replication (Hsiang et al., 2009; Zhao et al., 2010; Tang et al., 2010). In SARS-CoV-2, ISGylation of the N protein inhibits its oligomerization and reduces viral RNA synthesis (Rhamadianti et al., 2024).

To overcome ISGylation-mediated antiviral restriction, some viruses have evolved strategies to remove or neutralize ISG15 modifications, thereby restoring viral protein function and promoting replication. SARS-CoV-2 encodes the papain-like protease (PLpro), which specifically cleaves ISG15 from the N protein, relieving its functional inhibition, facilitating immune evasion, and enhancing viral replication (Zhu et al., 2024; Bang et al., 2024). Additionally, in recombinant IBV, although the NS1B protein does not directly inhibit the ISGylation process, it binds to and sequesters ISGylated NP protein, thereby blocking its antiviral function and indirectly alleviating ISGylation-mediated suppression of viral replication (Zhao et al., 2016). These examples illustrate how viruses can neutralize ISGylation without globally suppressing the host interferon response.

Notably, ISGylation does not universally restrict viral infection and can, in specific contexts, be repurposed to support viral replication. In HCV, the NS5A protein undergoes ISGylation at multiple lysine residues (K20, K26, K44, K139, and K166), a modification that promotes viral RNA replication (Bawono et al., 2021). Further studies using luciferase reporter replicon assays demonstrated that lysine 308 (K308) is critical for HCV replication (Abe et al., 2020). In this setting, ISGylation of NS5A functions as a proviral modification by recruiting the host factor cyclophilin A (CypA), thereby enhancing viral RNA replication.

## 10 Discussion

In this review, we synthesize recent evidence showing how 10 major post-translational modifications (PTMs) shape RNA virus infection across the viral life cycle, including entry, genome replication and transcription, particle assembly and release, and immune evasion (Table 1; Supplementary Tables 1–4). Although a wealth of studies has revealed the functions of diverse PTMs during viral infection, their precise regulatory mechanisms have yet to be fully elucidated.

As research progresses, increasing evidence suggests that different types of PTMs work synergistically to form complex regulatory networks that enable viruses to adapt to the dynamic host environment and achieve efficient infection. These modifications engage in a multi-layered and multidimensional interplay that balances immune defense and viral evasion, offering valuable insights into the intricate virus-host interactions. Early in infection, PTMs on viral surface and membrane-associated proteins often work together to promote entry and support productive infection: glycosylation shapes receptor binding and immune recognition, whereas lipidation enhances membrane association and trafficking and facilitates fusion-related steps. During the replication-transcription phase, the effect of a given PTM can be shaped by other modifications acting on the same protein. As infection progresses, viral proteins can shift between different modification states to coordinate the transition from genome replication to particle assembly and release. Placed along the infection timeline, these PTM events read as a coordinated sequence in which cooperative and competing modifications shape distinct functional states of viral proteins.

The host immune system dynamically modulates viral infection through PTMs, which not only act as important components of antiviral defense but also impose selective pressures that shape viral evolution. In viral proteins, PTM-targeted amino acid residues are frequently poorly conserved across strains, and even modifications at the same site can lead to distinct functional outcomes depending on the host cellular context. This pronounced variability highlights the context-dependent nature of PTM-mediated regulation and complicates efforts to generalize mechanistic insights across viruses or hosts. Addressing this complexity will require approaches that move beyond isolated modification events. In this regard, ongoing advances in proteomic and analytical approaches are progressively expanding the scope at which PTM regulation during viral infection can be examined (Li et al., 2025).

Viral replication is dependent on host-mediated PTMs that sustain the functional states of key viral proteins. In influenza A and B viruses, phosphorylation of a conserved S-S-S motif on the nuclear export protein is required for vRNP nuclear export and efficient replication, and disruption of this phosphorylation—via site mutation or inhibition of host kinases ATM/CK2—impairs viral replication in cells and animals; competitive blockade of this interaction with peptide mimics similarly lowers viral yield (Liu et al., 2025). These modification-dependent functional states of viral proteins therefore represent tractable points for antiviral targeting. Translating such PTM dependencies into therapeutic strategies, however, remains limited by host toxicity and imperfect specificity when targeting PTM enzymes, as well as by the context-dependent and dynamic nature of PTM states and their downstream interactions (Schor and Einav, 2018). Addressing these challenges will require deeper mechanistic understanding of how PTMs are established, coordinated, and functionally interpreted during viral infection.

Beyond the 10 PTMs class covered in this review, propelled by rapid advances in high-throughput modification omics technologies, in addition to classical PTMs such as phosphorylation and ubiquitination, emerging modifications including lactylation and ADP-ribosylation have been continuously discovered and confirmed to involved in virus infection and immune regulation mechanism. Among these, lactylation has emerged in the context of viral infection–associated metabolic reprogramming, with accumulating evidence linking lactylation to immune gene regulation, and occasional reports detecting lactylation on viral proteins in specific experimental systems (Chen et al., 2025). In contrast, ADP-ribosylation has been more firmly established as an antiviral modification, characterized by rapid induction during RNA virus infection and a conserved antagonism between host PARP enzymes and viral macrodomains (Zhang et al., 2025). Together, these emerging PTMs point to additional modes of regulation that operate alongside the canonical modification pathways and are only beginning to be defined in RNA virus infection. These developments are enabling more comprehensive and context-aware investigations of PTM function, which will be important for future studies aimed at clarifying how PTMs contribute to virus–host interactions.
