# Supplementary material for: The roles of post-translational modifications in the pathogenesis of RNA viruses: allies or adversaries?
Source: Front Microbiol. 2026 Feb 12;17:1768721. doi: 10.3389/fmicb.2026.1768721 (PMC12936031; doi:10.3389/fmicb.2026.1768721)
Supplement: Supplementary file 1 [file Table_1.DOCX]

**Supplementary Table 1 The Role of Ubiquitination and Ubiquitin-like Modifications in Viral Infection**

| Ubiqutination | **Virus** | **Protein** | **Site** | **Function** | **Reference PMID** |
| --- | --- | --- | --- | --- | --- |
|  | ZIKV | E | K38, K281 | Enhances viral attachment to the cell receptors，enhances binding to TIM-1 receptor, promoting viral entry and replication. | 32641828 |
|  | ZIKV | prM | - | Promotes viral release | 32574678 |
|  | IAV | M1 | K102, K104 | Accelerates M1 protein degradation, limiting viral replication. Facilitate dissociation of viral RNA from M1，mediating IAV release from the endosome. | 30328013, 24101521 |
|  | IAV | NP | K184, K227, K273 | Enhances viral replication by increasing RNA-binding affinity.  Ubiquitination of NP leads to protein degradation. | 20924359, 28536288, 29899090 |
|  | IAV | M2 | K78 | Facilitates the production of infectious viral particles | 29167343 |
|  | DENV | capsid protein | - | Promotes viral capsid degradation and genome uncoating | 27353759 |
|  | DENV | NS1 | K182, K189 | Reduces NS1’s ability to interact with its viral partner NS4B. | 29294313 |
|  | DENV | NS3 | K104 | Leads to NS3 ubiquitination and degradation. Modified by K27-linked polyubiquitin chains, promoting NS2B–NS3 interaction and enhancing NS2B3-mediated STING cleavage efficiency. | 30142214, 28228593 |
|  | CVB3 | 3D protein |  | Necessary for its function in controlling viral genome transcription. | 18612413 |
|  | HIV-1 | IN |  | Prevents integration of viral cDNA into the host genome. | 30804369 |
|  | HIV-1 | Gag |  | Disrupts viral particle release. | 11595185 |
|  | Ebola | VP35 |  |  | 28679761 |
|  | VSV | NP | - | Ubiquitination of NP leads to protein degradation | 31979016 |
|  | HCV | NS5B | K51 | Promotes the NS5B-NS5A interaction. | 33523994 |
|  | HCV | NS2 | - | Induces NS2-ESCRT interaction, promoting viral envelope formation | 30759391 |
|  | HCV | core protein | - | Protein ubiquitination and degradation,affect viral particle production | 17108031 |
|  | EV71 | 2C | K268 | Degrades the corresponding protein | 39212385 |
| SUMOylation | IAV | NS1 | K131, K217, K219, K221 | Enhances the stability of the NS1 protein | 21047957, 32822681 |
|  | IAV | M1 | K242 | Promotes M1-mediated viral RNP nuclear export and facilitates formation of the M1-vRNP complex | 25320310, 21507966 |
|  | DENV | NS5 | N-terminal | Enhances the stability of NS5 protein | 26889037 |
|  | HCV | NS5A | K348 | Enhances the stability of NS5A protein | 24602294 |
|  | EV71 | 3C | K52 | Reduces protein stability and enzymatic activity | 21784861 |
|  | EV71 | 3D | K159, 150-152 SIM | Enhances the stability of the viral polymerase | 27630238 |
|  | HIV-1 | IN | K46, K136, K244 | Regulates the affinity between IN and co-factors.  Inhibit the viral genome integration of HIV | 21454548, 22895527 |
|  | HIV-1 | p6 | K27 | Modulates HIV-1 infectivity | 15613319 |
|  | SARS-CoV-2 | NP | K62 | Enhances self-oligomerization and interferes with host cell division.  Facilitates viral RNP formation and nucleocapsid assembly | 15848177, 16998888 |
|  | HTLV | Tax | - | Regulate nuclear localization and trigger NF-κB signaling | 20959607 |
|  | ZIKV | NS5 | K252 | Disrupts host immune response | 30658479  32699085 |
| ISGylation | HCV | NS5A | - | Reduces the stability of NS5A protein | 20810994 |
|  | DENV-2 | NS3, NS5 | - | Inhibits viral particle release | 24769207 |

**Supplementary Table 2 The Role of Phosphorylation and Acetylation in Viral Infection**

| Phosphorylation | **Virus** | **Protein** | **Site** | **Function** | **Reference PMID** |
| --- | --- | --- | --- | --- | --- |
|  | IAV | NP | S9, Y10, T188, S165, S407, Y296, S269, S392 | Inhibiting the interaction between NP and importin-α to control the nucleocytoplasmic shuttling of NP. Inhibiting NES2-driven NP export, curtails vRNP polymerase activity and thereby attenuates influenza virus replication. Inhibiting NP oligomerization, suppresses RNP activity. Reduces the affinity between NP and CRM1, hindering RNP nuclear export. Promoting the NP-M1 interaction to enhance RNP nuclear export. | 25787277, 29775781, 25355893, 25867750, 25787277, 31440228, 32601201 |
|  | IAV | NS1 | S42, T80, T215, T49 | Weakening the NS1 protein's ability to bind dsRNA inhibits viral replication.  Attenuating NS1–NP interaction blocks vRNP transcription; disrupting NS1–RIG-I binding curbs interferon induction. Facilitating efficient viral replication.  Impairs NS1 binding to dsRNA, TRIM25, and RIG-I. | 22787231, 27376632, 19007960, 26687707 |
|  | IAV | M1 | Y132 | Promotes nuclear translocation of M1 and facilitates RNP nuclear export | 23536660 |
|  | IAV | PB1 | S478, T223, S673 | Inhibits viral transcription by blocking the NTP entry channel or interfering with RNA binding. | 32881973 |
|  | EBOV | VP35 | S187 | Facilitates efficient replication of the viral genome. | 31694758 |
|  | EBOV | VP30 |  | Promotes its binding to the nucleocapsid without affecting nucleocapsid transport or viral budding. | 35993739 |
|  | RV | capsid | S46 | Modulates the affinity for RNA binding. | 12525610 |
|  | YFV | NS5 | S56, T449 | Alters methyltransferase activity and inhibits viral translation.  Impacts viral replication. | 18757072,  19587048 |
|  | DENV | NS1 | T27, Y32 | Promotes RNA replication, NS1 dimerization, and production of infectious virus. | 34372598 |
|  | LASV | Z | Y97, S98 | Modulates viral particle release. | 30544850 |
|  | HIV-1 | p6 | T23, S40, S487 | Promotes viral particle formation.  Strengthens Gag-Vpr interaction to facilitate efficient Vpr incorporation into viral particles. | 15155723, 24257210, 24447338 |
|  | HTLV-1 | MA | S105 | Enhances the interaction between MA and the ESCRT complex, promoting viral budding and membrane scission. | 16635502 |
|  | SFV | NSP3 | - | Induces PI3K-Akt-mTOR activation and facilitates efficient internalization of the replication complex. | 26339054 |
| Acetylation | HIV | Tat | K28, K50, K51 | Activates Tat transcriptional activity. | 10545121, 11384967, 21220424 |
|  | HIV | IN | K258, K264, K266, K273 | Enhances IN-DNA binding affinity and promotes DNA strand transfer activity. | 16096645, 20226045 |
|  | SRAS-CoV-2 | N | Multiple sites | Modulates N protein-RNA binding affinity. | 33894414 |
|  | HDV | S-HDAg | K72 | Regulates nucleocytoplasmic shuttling of the viral genome. | 14967488 |
|  | IAV | NP | K103，K31, K90，K77, K113, K229 | HDAC1 removes NP acetylation, enhancing RNP activity.  Regulates RNP activity.  Modifies RNP activity; modification at K229 inhibits viral particle release. | 29312300, 29555684,  29097654 |
|  | IAV | PA | K19, K664 | Enhances PA endonuclease and viral RNA-dependent RNA polymerase activities.  Deacetylation at this site inhibits viral replication. | 34270849, 30518648 |
|  | IAV | NS1 | K108 | Enhances NS1 protein’s IFN-β antagonistic activity. | 32093780 |
|  | Ebov | NP | - | Affects NP-RNA interaction. | 30205953 |
|  | Ebov | VP40 | K274 | Regulates VP40–lipid bilayer interaction, affecting viral budding efficiency | 30205953 |

**Supplementary Table 3 The Role of Palmitoylation and Myristoylation in Viral Infection**

| Palmitoylation | **Virus** | **Protein** | **Site** | **Function** | **Reference PMID** |
| --- | --- | --- | --- | --- | --- |
|  | HCV | core protein | C172 | Stabilizes core protein association with the endoplasmic reticulum membrane | 19783655 |
|  | SFV | NSP1 | C418-C420 | Enhances protein association with the cytoplasmic membrane | 10888610 |
|  | Sindbis Virus | TF | - | Selectively transported to the plasma membrane for viral budding | 27852864 |
|  | SARS-CoV-2 | E | - | Increases cell membrane permeability | 16507314 |
|  | HIV-1 | gp160 | C764, C837 | Promotes gp160 anchoring to lipid rafts | 11095714 |
|  | SARS-CoV-2 | S | - | Required for DRM localization and membrane fusion | 20580052,19801669 |
|  | IAV | HA | - | Positively correlates with viral particle assembly | 16227287 |
| Myristoylation | LASV | Z | - | Necessary for Z protein binding to membranes | 17083745,15452271 |
|  | EV71 | VP4 | - | Regulates the release of viral genomic RNA from the capsid into the cytoplasm | 32399947 |
|  | HIV | Nef |  | Activates NF-κB and AP-1, leading to enhanced viral replication | 18336259 |

**Supplementary Table 4 The Role of Glycosylation in Viral Infection**

| Glycosylation | **Virus** | **Protein** | **Site** | **Function** | **Reference PMID** |
| --- | --- | --- | --- | --- | --- |
|  | DENV | NS1 | N130, N207 | Enhances NS1 protein’s cell surface expression, secretion, stability, and function. | 21429549 |
|  | HTNV | Gn 、Gc | N134, N235, N347, N399, N928 | Promotes protein folding and intracellular trafficking. | 15113920 |
|  | NiV | F | - | Reduces viral fusion and entry efficiency, “protecting” the virus from neutralizing antibodies | 16641279 |
|  | NiV | G | - | Reduces viral fusion efficiency. | 22915812 |
|  | Hendra virus | F | N67, N99, N414, N464 | Promotes viral fusion by ensuring proper folding, maturation, and membrane localization of the F protein | 15919949 |
|  | HCV | E1, E2 | - | Facilitates folding and heterodimerization of E1 and E2 proteins.  Stabilizes envelope proteins and promotes viral assembly.  Facilitates HCV evasion of humoral immunity | 15956584, 20844034, 17522218 |
|  | ZIKV | E | N154 | Enhances viral virulence. | 29091758,30944176 |
|  | RRV | E1 | - | Enhances viral virulence. | 26813162 |
|  | DENV-2 | E | N67 | Facilitates viral particle assembly and release. | 17459925,17543367 |
|  | IAV | HA | - | Prevented the binding of antibodies to Antigenic sites | 22258255 |
|  | HCoV-NL63 | S | N358 | Glycan shield obstructing | 27617430 |
|  | LASV | GP-1 | - | Impairs the protective efficacy of neutralizing antibody | 26587982 |
|  | HIV-1 | Env | - | Affects viral infectivity and sensitivity to monoclonal antibody neutralization. | 23384254 |
